# Body image and maladaptive eating behaviors in Chinese early adolescents: a latent profile analysis

**DOI:** 10.3389/fpsyg.2026.1879943

**Published:** 2026-10-02

**Authors:** Chungan Zhang, Yichu Han, Shuhua Cheng, Yuqian Qi

**Affiliations:** 1 School of Educational Science, Harbin Normal University, Harbin, China; 2 College of Education, Huzhou Normal University, Huzhou, China; 3 School of Teacher Education, Qiqihar University, Qiqihar, China

**Keywords:** body dissatisfaction, early adolescence, latent profile analysis, maladaptive eating behaviors, positive body image

## Abstract

**Background:**

Body image undergoes significant changes during early adolescence, yet empirical evidence on how the combination of negative and positive body image in this stage relates to maladaptive eating behaviors remains limited. To address this, employing a person-centered approach, this study aimed to identify distinct profiles of body image and to explore the relationships between profile membership, gender, body mass index (BMI), socioeconomic status (SES), and maladaptive eating behaviors among Chinese early adolescents.

**Methods:**

Using indicators from three dimensions of positive body image (i.e., body self-appreciation, body other-appreciation, resilience against negative appearance feedback) and negative body image (i.e., body dissatisfaction), latent profile analysis (LPA) was conducted with a sample of 1,248 junior high school students (aged 12–15 years, *M* = 13.42, *SD* = 1.03; 50.4% boys) from Heilongjiang Province, China.

**Results:**

Three distinct body image profiles were identified: the Positive Body Image Group (66.59%), the Body Other-Appreciation Dominant Group (23.88%), and the Moderate Body Image Group (9.54%). Gender and BMI significantly predicted profile membership, whereas SES did not. Regarding maladaptive eating behaviors, both the Body Other-Appreciation Dominant Group and the Moderate Body Image Group reported significantly higher levels of restrained eating than the Positive Body Image Group. The Moderate Body Image Group also reported significantly higher levels of emotional eating compared with the Positive Body Image Group. No significant differences were found in external eating among the three profiles.

**Conclusion:**

Early adolescents' body image profiles exhibit significant heterogeneity, with distinct patterns of association with maladaptive eating behaviors across profiles. Accordingly, interventions should consider gender and body mass index and be tailored to the characteristics of each profile to improve body image and eating behaviors.

## 1 Introduction

Body image is a multidimensional psychological construct defined as an individual's self-perceptions and self-attitudes toward their own body, particularly (though not exclusively) its appearance, encompassing thoughts, beliefs, feelings, and behaviors (Cash, 2004). Body image can be extended into positive body image and negative body image. Positive body image is a multifaceted construct that includes love, respect, acceptance, and appreciation of one's body, a sense of harmony with the body, and the ability to process body-related information in a protective manner (Tylka and Wood-Barcalow, 2015b; Wood-Barcalow et al., 2010). In contrast, negative body image is characterized by disrespect, dissatisfaction, or shame toward the body (Gattario and Frisén, 2019). Historically, research has predominantly focused on the negative aspects of body image and their links to pathological outcomes (Tylka, 2011). However, with the introduction of the positive psychology perspective, scholars increasingly recognize that positive body image is not merely the opposite or absence of negative body image. Solely targeting the reduction of negative body image symptoms while neglecting the cultivation and promotion of positive body image may not only constrain an individual's capacity to flourish in body-related experiences (Cash and Smolak, 2011) but also undermine the effectiveness of prevention and intervention efforts for body image disturbances and related issues (Tylka and Wood-Barcalow, 2015b). Therefore, integrating both positive and negative body image within a unified framework is essential for advancing a more comprehensive understanding of individual body image development and for designing effective interventions.

During early adolescence, body image undergoes significant changes, with most individuals exhibiting a sharp increase in negative body image levels (Bucchianeri et al., 2013; Eisenberg et al., 2006), which tend to stabilize only in adulthood (Grogan, 2017). According to the Tripartite Influence Model (Thompson et al., 1999), this developmental change is driven by sociocultural pressures from family, peers, and media, which are transmitted through the mediating processes of appearance ideal internalization and social comparison—mechanisms that have been empirically documented among early adolescents (Keery et al., 2004; Shroff and Thompson, 2006). Concurrently, rapid physical maturation during this stage, such as weight gain and alterations in body shape, may intensify body dissatisfaction among them (Voelker et al., 2015). Given the convergence of these psychological, sociocultural, and biological factors, early adolescence constitutes a critical window for the development of body image disturbances. Therefore, it is essential to identify body image concerns in early adolescence and implement prevention and intervention efforts, with the aim of fostering positive outcomes throughout adolescence and into adulthood.

Body image is associated with various health outcomes, among which eating disorders represent a particularly detrimental consequence. Specifically, negative body image is widely recognized as a key risk and maintenance factor for the development and persistence of eating pathology (Stice and Shaw, 2002). Maladaptive eating behavior, situated between adaptive eating and clinically diagnosed eating disorders, constitutes a subclinical state that does not fully meet diagnostic criteria for an eating disorder (Franko and Omori, 1999; Qian and Liu, 2002). Such behavior is primarily categorized into three types based on distinct eating motives: restrained eating, emotional eating, and external eating (van Strien et al., 1986). Restrained eating refers to the tendency to diet in order to reduce or maintain a specific body weight (Tuschl, 1990). Emotional eating describes the tendency to eat in response to both positive and negative emotions (Turton et al., 2017). External eating refers to eating in response to external food cues (e.g., seeing or smelling palatable food), independent of internal hunger signals (Nisbett, 1968). A substantial body of research consistently demonstrates that negative body image among adolescents positively predicts their maladaptive eating behaviors (Evans et al., 2019; Gerner and Wilson, 2005; Shriver et al., 2020). Longitudinal studies have further revealed that maladaptive eating behaviors and negative body image mutually reinforce each other among adolescents, creating a vicious cycle (Liu et al., 2023; Zhang et al., 2023b). In contrast, positive body image serves as a protective factor against eating problems and can promote adaptive eating behaviors (Linardon et al., 2022). Positive and negative body image are not necessarily mutually exclusive; they may coexist within the same individual (Iannattone et al., 2025a; Tylka and Wood-Barcalow, 2015b), potentially exerting distinct influences on eating behaviors (Jackson et al., 2022). However, how this coexistence relates to maladaptive eating behaviors in early adolescence remains unclear.

Previous research has predominantly employed a variable-centered approach to examine the relationship between positive and negative body image, largely overlooking the possibility of their coexistence. In recent years, however, a number of studies have adopted a person-centered approach, utilizing both positive and negative body image as indicators to identify distinct subgroups characterized by specific combinations of body image attributes within various populations. These studies further explore the associated predictors and outcomes of such subgroup memberships (Baker et al., 2023; Iannattone et al., 2025a,b; Jackson et al., 2022; More et al., 2024; Raspovic et al., 2022, 2023). For instance, Jackson et al. (2022) conducted a Latent Profile Analysis (LPA) using body appreciation, functional appreciation, body dissatisfaction, and weight bias internalization as indicators. They simultaneously examined the relationship between the identified body image latent profiles and eating behaviors. Their findings revealed four profiles among U.S. adults: “appreciation and mild dissatisfaction,” “functional appreciation and moderate dissatisfaction,” “strong dissatisfaction,” and “strong appreciation.” The “strong appreciation” profile exhibited the lowest scores on maladaptive eating behaviors. Similarly, Iannattone et al. (2025a), using body appreciation, functional appreciation, body dissatisfaction, and body shame as indicators, identified three consistent profiles via LPA for both Italian adolescent boys and girls: “Strong negative body image and weak positive body image,” “Weak negative body image and strong positive body image,” and “Moderate negative and positive body image.” The “Moderate negative and positive body image” profile was the most prevalent. Collectively, these two studies demonstrate that positive and negative body image can coexist within certain subgroups of both adults and adolescents. The finding that a substantial proportion of adolescents belong to a profile characterized by moderate levels on both indicators may further reflect ambivalent or contradictory self-perceptions regarding their bodies.

However, LPA research in general has predominantly used self-directed indicators of positive body image (e.g., body appreciation, functional appreciation), and has not yet incorporated other-directed indicators, such as the appreciation of others' bodies. Notably, qualitative research with early adolescents has provided evidence for this other-directed form of appreciation. Holmqvist and Frisén (2012) found that early adolescents with high body esteem held broad and flexible definitions of beauty, criticized narrow media ideals, and expressed appreciation for diverse bodies. Building on this foundation, Maes et al. (2021) identified a four-dimensional framework of adolescent positive body image: (a) body self-appreciation, defined as the acceptance, respect, and appreciation of one's own body; (b) body other-appreciation, which refers to the acceptance, respect, and appreciation of others' bodies, a concept grounded in a broad conceptualization of beauty that posits adolescents embrace diverse body standards and perceive all bodies as beautiful; (c) resilience against body ideals in media, representing the capacity to withstand unrealistic beauty standards conveyed by media outlets; and (d) resilience against negative appearance feedback, reflecting the capacity to cope positively with negative body-related information. Although this multidimensional framework offers a more comprehensive conceptualization of positive body image, it remains unclear how multidimensional positive body image coexists with negative body image, and no study has yet applied this framework to person-centered research among Chinese early adolescents.

From a developmental perspective, early adolescence is an important stage for examining this coexistence. A longitudinal meta-analysis demonstrated that the magnitude of change in body image during early adolescence is the largest across the lifespan (Lacroix et al., 2023). This period is also characterized by dramatic physiological, cognitive, emotional, and social changes, which shape adolescents' body-related self-perceptions and attitudes. Meanwhile, in the Chinese cultural context, most LPA studies have examined body image classification using only positive body image (Yan, 2025; Zhang et al., 2023a) or negative body image (Guo et al., 2025; Li et al., 2024; Xu et al., 2025a, 2023, 2025b) as indicators. Investigation into the potential coexistence of these two constructs has been extremely limited, with only studies involving pregnant women providing preliminary evidence (Zhao et al., 2025a,b). Therefore, examining whether similar or unique profiles emerge among Chinese early adolescents is a necessary extension of this line of inquiry.

### 1.1 Current study

Building on the existing research landscape outlined above, we employed LPA to identify distinct subgroups among Chinese early adolescents, using positive body image (based on the multidimensional structure established by Maes et al., 2021) and negative body image (i.e., body dissatisfaction) as indicators. Concurrently, considering that gender (Rierdan et al., 1988), body mass index (BMI; Latiff et al., 2018), and socioeconomic status (SES; van den Berg et al., 2010) have been established as correlates of early adolescent body image, we examined these three factors as predictors of profile membership. Finally, we investigated the associations between the identified body image profiles and three distinct maladaptive eating behaviors: restrained eating, emotional eating, and external eating.

Specifically, we aim to address three research questions: (1) What distinct body image profiles exist among Chinese early adolescents, and is there a profile characterized by the coexistence of positive and negative body image? (2) Do gender, BMI, and SES predict profile membership? (3) Are the identified profiles associated with maladaptive eating behaviors? Given the exploratory nature of latent profile analysis, the limited direct research on the coexistence of positive and negative body image among early adolescents, and the fact that previous person-centered studies have predominantly used unidimensional measures of positive body image that do not include dimensions such as body other-appreciation, we did not formulate specific directional hypotheses.

## 2 Method

### 2.1 Participants

A total of 1,350 students from three junior high schools in Heilongjiang Province, China, were invited to participate through cluster sampling. Data were collected via paper-based questionnaires administered in classroom settings. After excluding incomplete and invalid responses, 1,248 valid questionnaires were retained, resulting in a valid response rate of 92.44%. The participants had a mean age of 13.42 years (*SD* = 1.03; range = 12–15). The sample consisted of 629 boys and 619 girls. By grade level, there were 490 students in Grade 7, 470 in Grade 8, and 288 in Grade 9. This study received approval from the Ethics Committee of the School of Educational Science, Harbin Normal University and was carried out in accordance with the guidelines and regulations of this committee.

### 2.2 Measures

#### 2.2.1 Positive body image

Positive body image was assessed using the Positive Body Image among Adolescents Scale (PBIAS; Maes et al., 2021). With permission from Maes, the scale was translated into Chinese. This translation process incorporated feedback from a professor of English and involved discussions within a team of psychological experts and graduate students. The original 15-item scale was reduced to 10 items through the following steps. After consultation with junior high school students and psychological experts, the two items of the “resilience against body ideals in media” dimension were deleted due to their low comprehensibility and limited alignment with the media experiences of Chinese early adolescents. Subsequently, three additional items with cross-loadings or inadequate loadings were removed following exploratory factor analysis (EFA). The retained three-factor model (body self-appreciation, body other-appreciation, and resilience against negative appearance feedback) was validated through confirmatory factor analysis, which demonstrated good model fit: χ^2^/*df* = 4.16, RMSEA = 0.05, SRMR = 0.04, CFI = 0.97, TLI = 0.95. To assess criterion-related validity, we examined the correlations between PBIAS scores and scores on the Body Appreciation Scale-2 (BAS-2; Tylka and Wood-Barcalow, 2015a). PBIAS total scores were significantly and positively correlated with BAS-2 total scores (*r* = 0.69, *p* < 0.001), as were its three subscales (*rs* = 0.49–0.66, *ps* < 0.001), supporting the scale's criterion-related validity. Items are rated on a 7-point Likert scale (from strongly disagree to strongly agree), with higher scores indicating a higher level of positive body image. In the present study, the Cronbach's alpha coefficient for the PBIAS was 0.86.

#### 2.2.2 Body dissatisfaction

Body dissatisfaction was assessed using the General Appearance subscale of the Negative Physical Self Scale (NPS; Chen et al., 2006). The scale comprises 5 items, all of which are reverse-scored. Responses are provided on a 5-point Likert scale ranging from never to always. Higher scores indicate a higher level of body dissatisfaction, corresponding to a more negative body image. In the present study, the Cronbach's alpha coefficient for this scale was 0.88.

#### 2.2.3 Maladaptive eating behaviors

Maladaptive eating behaviors were assessed using the Dutch Eating Behavior Questionnaire (DEBQ; van Strien et al., 1986). This questionnaire comprises 33 items across three subscales: restrained eating, emotional eating, and external eating. Responses are recorded on a 5-point Likert scale ranging from never to always. Higher scores on each subscale indicate a greater level of the respective maladaptive eating behavior. In the present study, the Cronbach's alpha coefficients for the Restrained Eating, Emotional Eating, and External Eating subscales were 0.89, 0.97, and 0.90.

#### 2.2.4 BMI and SES

BMI was calculated as weight in kilograms divided by height in meters squared (kg/m^2^). Given the developmental age range of our sample (12–15 years), we used age- and sex-adjusted BMI z-scores to account for growth-related changes in BMI during adolescence. Specifically, BMI z-scores were calculated based on the Chinese national reference standards for children and adolescents aged 0–18 years (Li et al., 2009). Socioeconomic status SES was derived from three indicators: parental education level, parental occupational status, and monthly household income. Parental education was scored on a scale of 1 to 6, corresponding to the following levels: 1 = primary school or below, 2 = junior high school, 3 = senior high school or technical secondary school, 4 = junior college, 5 = undergraduate degree, 6 = postgraduate degree (master's or doctoral). The higher score of either parent was used. Parental occupational status was coded into five levels [adapted from Shi and Shen (2007)]: 1 = temporary workers, unemployed, jobless individuals, and farmers; 2 = manual laborers, small business owners, and ordinary workers; 3 = skilled workers, commercial/service employees, and clerical staff; 4 = professionals (e.g., teachers, doctors, lawyers, engineers); 5 = senior managers in enterprises or institutions, senior professionals, leaders and staff in government agencies, and private business owners. The higher level from either parent was taken. Monthly household income was scored from 1 to 5: 1 = 4,000 RMB or less, 2 = 4,001–6,000 RMB, 3 = 6,001–8,000 RMB, 4 = 8,001–10,000 RMB, 5 = 10,001 RMB or more. After standardizing these three indicators, a composite SES score was derived using factor analysis [following Ren (2010)]. Specifically, the first principal factor was extracted, and the SES score was calculated as the sum of the standardized indicators weighted by their respective factor loadings, divided by the eigenvalue of the first principal factor.

### 2.3 Data analysis

Data analysis was conducted using SPSS 29.0 (IBM Corporation, Armonk, New York, USA) and Mplus 8.3 (Muthén & Muthén, Los Angeles, California, USA). A one-way analysis of variance (ANOVA) was conducted with SPSS 29.0. LPA was carried out in Mplus 8.3, using the three dimensions of positive body image (i.e., body self-appreciation, body other-appreciation, and resilience against negative appearance feedback) along with body dissatisfaction as continuous indicator variables. To determine the optimal number of profiles, we sequentially tested models with one to five profiles, and the final solution was selected based on a comprehensive evaluation of statistical fit indices and theoretical interpretability. Model fit was evaluated using the following indices: log likelihood (LL), Akaike information criterion (AIC), Bayesian information criterion (BIC), sample size-adjusted Bayesian information criterion (aBIC), entropy, the Lo-Mendell-Rubin likelihood ratio test (LMR-LRT), and the bootstrap likelihood ratio test (BLRT). Lower values of AIC, BIC, and aBIC indicate better model fit (Nylund et al., 2007). Higher entropy values indicate more precise classification, with values above 0.80 generally considered acceptable (Muthén, 2001). Significant *p* values for the LMRT and BLRT suggest that the k-profile model fits the data better than the (*k*−1)-profile model (Tein et al., 2013). Furthermore, the smallest class proportion should typically exceed 5% of the sample (Nylund-Gibson and Choi, 2018). After identifying the optimal model based on these criteria, the R3STEP method (Asparouhov and Muthén, 2014) was employed to examine the effects of predictors (gender, BMI, and SES) on the distinct latent profile membership. Subsequently, the BCH method (Bakk and Vermunt, 2016) was used to investigate the association between these profiles and the continuous outcomes (maladaptive eating behaviors).

## 3 Results

### 3.1 Descriptive statistics and correlations

Descriptive statistics and correlations among the study variables are presented in Table 1. Restrained eating was negatively correlated with all three positive body image dimensions (*rs* = −0.09 to −0.19, *ps* < 0.001) and positively correlated with body dissatisfaction (*r* = 0.19, *p* < 0.001). Emotional eating was negatively correlated with body other-appreciation (*r* = −0.09, *p* < 0.01) and body self-appreciation (*r* = −0.07, *p* < 0.05), but was not significantly correlated with resilience against negative appearance feedback or body dissatisfaction (*ps* > 0.05). External eating was not significantly correlated with any of the positive body image dimensions or body dissatisfaction (*ps* > 0.05).

**Table 1:** Descriptive statistics and correlations among variables.

Variable	1	2	3	4	5	6	7
1. PBIAS1	1						
2. PBIAS2	0.55^***^	1					
3. PBIAS3	0.51^***^	0.40^***^	1				
4. BD	−0.66^***^	−0.41^***^	−0.50^***^	1			
5. DEBQ1	−0.19^***^	−0.09^***^	−0.14^***^	0.19^***^	1		
6. DEBQ2	−0.09^**^	−0.07^*^	−0.01	0.02	0.21^***^	1	
7. DEBQ3	−0.03	0.00	0.01	−0.04	0.08^**^	0.56^***^	1
*M*	6.51	5.86	5.35	2.20	2.47	1.95	2.92
*SD*	0.96	1.19	1.54	0.96	1.01	1.08	0.96

PBIAS1, Body other-appreciation; PBIAS2, Body self-appreciation; PBIAS3, Resilience against negative appearance feedback; BD, Body dissatisfaction; DEBQ1, Restrained eating; DEBQ2, Emotional eating; DEBQ3, External eating. ^*^p < 0.05, ^**^p < 0.01, ^***^p < 0.001.

### 3.2 LPA of body image among early adolescents

To identify the latent body image profiles among early adolescents, LPA was conducted. Models with one to five latent profiles were estimated sequentially. As presented in Table 2, the AIC, BIC, and aBIC values decreased consistently as the number of profiles increased. Entropy values for the 2- to 5-profile models were all above the recommended threshold of 0.80, indicating adequate classification accuracy. The BLRT was significant for each stepwise model comparison (*p* < 0.05). The LMR-LRT was significant for the 2-, 3-, and 4-profile models (*p* < 0.05), but became non-significant when testing the 5-profile model against the 4-profile solution (*p* > 0.05). Furthermore, one of the latent profiles in the 4-profile model comprised less than 5% of the sample, which is below the recommended minimum proportion for profile interpretability and stability. Although the 2-profile model was statistically acceptable, it was deemed too parsimonious to capture the nuanced heterogeneity in the data. Considering the statistical evidence, theoretical meaningfulness, and practical interpretability collectively, the 3-profile solution was retained as the optimal model.

**Table 2:** LPA of body image among early adolescents.

Model	NP	LL	AIC	BIC	aBIC	*p*LMR-LRT	*p*BLRT	Entropy	Smallest profile (%)
1P	8	−7,742.54	15,501.08	15,542.11	15,516.70				100
2P	13	−6,910.29	13,846.59	13,913.27	13,871.97	< 0.001	< 0.001	0.97	11.78
3P	18	−6,569.31	13,174.63	13,266.95	13,209.78	< 0.001	< 0.001	0.86	9.54
4P	23	−6,432.49	12,910.98	13,028.95	12,955.89	< 0.001	< 0.001	0.86	3.21
5P	28	−6,232.42	12,520.85	12,664.47	12,575.53	0.16	< 0.001	0.90	1.60

NP, Number of free parameters in the model; LL, Log-likelihood.

Based on the LPA results, the three profiles were described based on their scores on the three positive body image dimensions and body dissatisfaction. One-way ANOVAs were conducted to examine differences across the profiles on these indicators, with results presented in Table 3. Profiles were labeled based on how their scores differed from each other and how the dimensions related to each other within each profile (see Figure 1). Specifically, Profile 3 (66.59%) was characterized by high scores on all positive body image dimensions and a low score on body dissatisfaction. It was therefore designated the Positive Body Image Group. Compared to Profile 3, both Profile 1 (23.88%) and Profile 2 (9.54%) scored lower on all positive body image dimensions and higher on body dissatisfaction. In particular, within Profile 1, the score on the body other-appreciation dimension was higher than its scores on the other two positive body image dimensions. Consequently, this profile was labeled the Body Other-Appreciation Dominant Group. In contrast, Profile 2 exhibited moderate levels across all indicators and was thus named the Moderate Body Image Group.

**Table 3:** Means, standard deviations, and one-way ANOVA results for body image indicators across latent profiles.

Variables	P1 (*n* = 298) (*M* ±*SD*)	P2 (*n* = 119) (*M* ±*SD*)	P3 (*n* = 831) (*M* ±*SD*)	*F*	*η^2^*	Bonferroni
Body self-appreciation	4.85 ± 0.97	3.97 ± 1.18	6.49 ± 0.57	920.25^***^	0.60	P3>P1>P2
Body other-appreciation	6.50 ± 0.56	3.99 ± 0.92	6.88 ± 0.34	1,879.27^***^	0.75	P3>P1>P2
Resilience against negative appearance feedback	4.32 ± 1.44	3.66 ± 1.58	5.96 ± 1.13	307.50^***^	0.33	P3>P1>P2
Body dissatisfaction	3.11 ± 0.60	3.31 ± 0.83	1.72 ± 0.68	632.89^***^	0.50	P2>P1>P3

^*^p < 0.05, ^**^ *p* < 0.01, ^***^ *p* < 0.001. P1, Body Other-Appreciation Dominant Group; P2, Moderate Body Image Group; P3, Positive Body Image Group. *Post hoc* comparisons were conducted using the Bonferroni correction.

**Figure 1:**
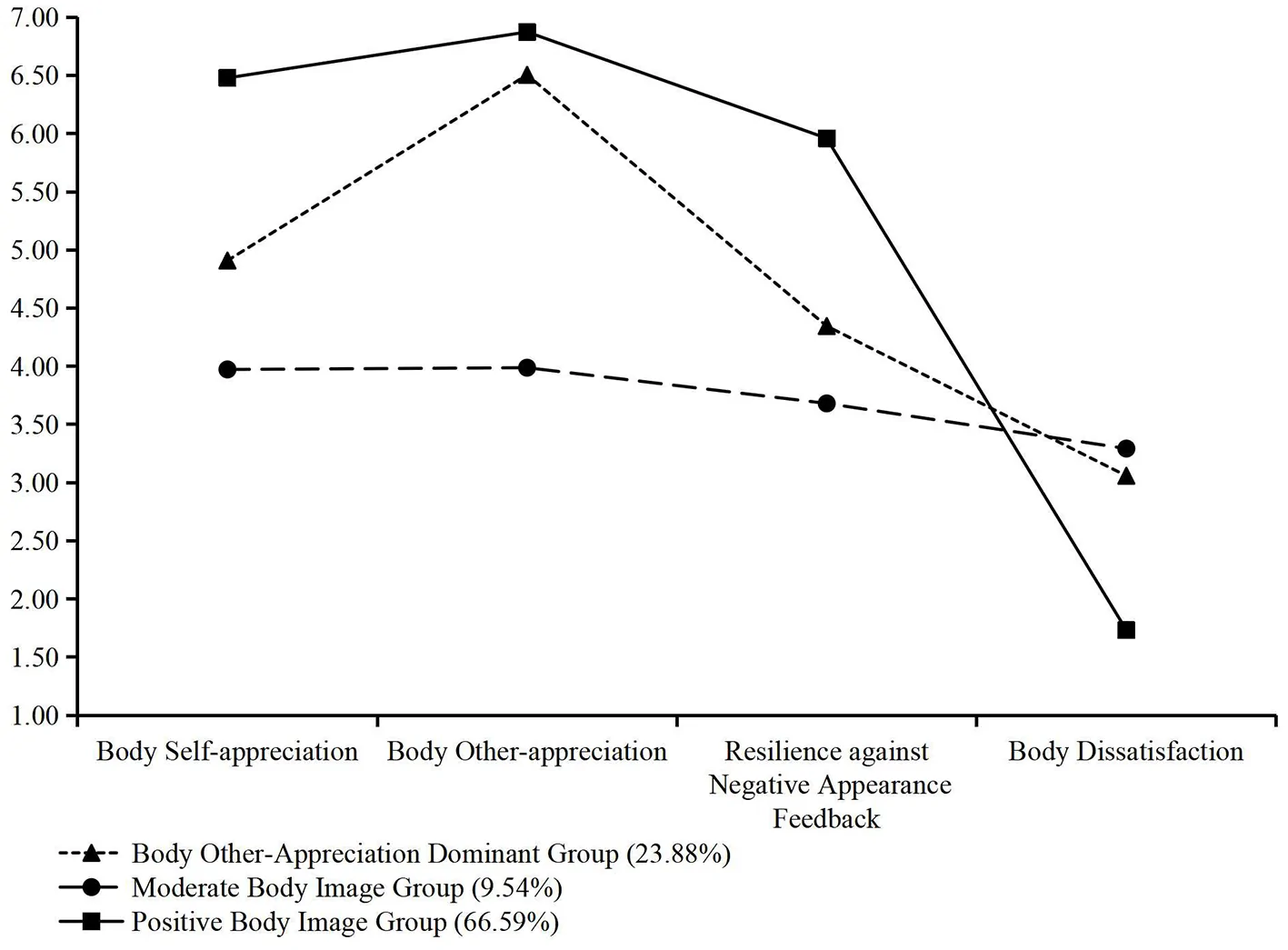
Results of the 3-profile body image model among early adolescents.

### 3.3 Predictors of body image profiles: gender, BMI, and SES

To examine the effects of gender (0 = boys, 1 = girls), BMI, and SES on body image profile membership, the R3STEP method was employed (Asparouhov and Muthén, 2014), using the Positive Body Image Group as the reference profile (see Table 4). The results indicated that gender (β = 0.67, *p* < 0.001) and BMI (β = 0.28, *p* < 0.001) significantly predicted latent profile membership, whereas SES was not a significant predictor (*p* > 0.05). Specifically, compared to boys, girls had 96% higher odds of belonging to the Body Other-Appreciation Dominant Group relative to the Positive Body Image Group (*p* < 0.001). Furthermore, for each one-unit increase in BMI, the odds of belonging to the Body Other-Appreciation Dominant Group (vs. the Positive Body Image Group) increased by 32% (*p* < 0.001). Gender and BMI did not significantly distinguish the Moderate Body Image Group from the Positive Body Image Group.

**Table 4:** Predictors of profile membership vs. the positive body image group (R3STEP).

Predictors	Body other-appreciation dominant group			Moderate body image group		
	β	*SE*	*OR* 95% *CI*	β	*SE*	*OR* 95% *CI*
Gender	0.67^***^	0.19	1.96 (1.36, 2.82)	−0.07	0.25	0.94 (0.58, 1.52)
BMI	0.28^***^	0.07	1.32 (1.16, 1.51)	0.10	0.09	1.11 (0.92, 1.33)
SES	−0.05	0.09	0.95 (0.80, 1.13)	−0.17	0.12	0.85 (0.67, 1.07)

^*^p < 0.05, ^**^ *p* < 0.01, ^***^ *p* < 0.001.

### 3.4 Associations of body image profiles with maladaptive eating behaviors

The BCH method (Bakk and Vermunt, 2016) was employed to examine the associations between body image profiles and maladaptive eating behaviors (see Table 5). The results indicated significant overall differences across the three latent body image profiles for both restrained eating (χ^2^ = 38.94, *p* < 0.001), and emotional eating (χ^2^ = 6.19, *p* < 0.05). No significant overall difference was found for external eating (*p* > 0.05). Specifically, early adolescents in both the Body Other-Appreciation Dominant Group (χ^2^ = 26.93, *p* < 0.001), and the Moderate Body Image Group (χ^2^ = 19.72, *p* < 0.001), reported significantly higher levels of restrained eating than those in the Positive Body Image Group. For emotional eating, early adolescents in the Body Other-Appreciation Dominant Group (χ^2^ = 4.08, *p* < 0.05), and the Positive Body Image Group (χ^2^ = 6.17, *p* < 0.05), reported significantly lower levels than those in the Moderate Body Image Group.

**Table 5:** Associations between body image profiles and maladaptive eating behaviors (BCH).

Outcomes	Profile	*M*	*SE*	Comparison	*χ^2^*	Overview
Restrained eating	P1	2.72	0.06	Overall test	38.94^***^	P1>P3; P2>P3
	P2	2.76	0.09	P1 vs. P2	0.16	
	P3	2.33	0.04	P1 vs. P3	26.93^***^	
				P2 vs. P3	19.72^***^	
Emotional eating	P1	1.94	0.07	Overall test	6.19^*^	P1 < P2; P2>P3
	P2	2.21	0.11	P1 vs. P2	4.08^*^	
	P3	1.92	0.04	P1 vs. P3	0.09	
				P2 vs. P3	6.17^*^	
External eating	P1	2.92	0.06	Overall test	0.06	
	P2	2.94	0.09	P1 vs. P2	0.04	
	P3	2.92	0.04	P1 vs. P3	0.00	
				P2 vs. P3	0.06	

^*^p < 0.05, ^**^ *p* < 0.01, ^***^ *p* < 0.001. P1, Body Other-Appreciation Dominant Group; P2, Moderate Body Image Group; P3, Positive Body Image Group.

## 4 Discussion

We employed LPA to identify distinct profiles of body image in early adolescence, using three dimensions of positive body image (i.e., body self-appreciation, body other-appreciation, and resilience against negative appearance feedback) and body dissatisfaction as indicators. Furthermore, we investigated whether gender, BMI, and SES predicted membership in the identified latent profiles, and examined the associations between profile membership and maladaptive eating behaviors.

The LPA results revealed three distinct latent profiles: the Body Other-Appreciation Dominant Group, the Moderate Body Image Group, and the Positive Body Image Group. First, the Positive Body Image Group constituted the largest proportion of the sample. This profile was characterized by high levels of positive body image and low levels of negative body image, with significantly higher positive body image scores and lower negative body image scores compared to the other two profiles. This suggests that the majority of Chinese early adolescents in this sample exhibit a positive body image profile, a finding consistent with prior LPA research on negative body image among Chinese adolescents (e.g., Xu et al., 2023), which identified a “body satisfaction” profile comprising 65.2% of participants. Second, the Body Other-Appreciation Dominant Group represented the second-largest profile. Its defining feature was a higher level of body other-appreciation relative to levels of body self-appreciation and resilience against negative appearance feedback. This profile revealed a contradictory state in body image, wherein individuals can accept and appreciate diverse body types in others but struggle to extend this appreciation to themselves. This discrepancy may indicate an incomplete internalization of a broad conceptualization of beauty, which posits that genuine body appreciation should be directed toward both others and the self (Tylka and Iannantuono, 2016). For early adolescents, this asynchronous state of self- vs. other-directed appreciation warrants attention, as it represents an incomplete form of positive body image. Without a foundation of body self-appreciation, such body other-appreciation may undermine their wellbeing (Maes et al., 2021). Third, the Moderate Body Image Group was the smallest profile. It was characterized by moderate levels of both positive and negative body image. This finding corroborates the potential for a state of “normative ambivalence” in adolescent body image (Iannattone et al., 2025a), where positive and negative dimensions coexist. It aligns with previous LPA studies (Iannattone et al., 2025a,b; Jackson et al., 2022; Raspovic et al., 2022, 2023; Zhao et al., 2025a,b) and further validates this coexistence within a Chinese early adolescent sample. Notably, the finding that this profile was the least prevalent contrasts with results from Iannattone et al. (2025a), who found it to be the most common profile among Italian adolescents. This discrepancy may reflect cultural differences and suggests that the prevalence of this profile may fluctuate significantly across developmental stages, a possibility requiring further investigation. In addition to these three identified profiles, another profile commonly reported in LPA literature, described as the “strong negative body image and weak positive body image”, has been identified in studies of adolescents (Iannattone et al., 2025a), adults (e.g., Jackson et al., 2022; Raspovic et al., 2023), and within Chinese samples focusing on pregnant women (Zhao et al., 2025a,b). Given that negative body image typically peaks during adolescence (Ata et al., 2007), the potential existence of this high-risk subgroup in early adolescents should not be overlooked. Its absence in the present sample may reflect the developmental characteristics of early adolescence, suggesting that the most extreme form of body image disturbance may emerge later in development. This possibility warrants further examination in future research.

The present study found that gender and BMI significantly predicted latent profile membership of body image among early adolescents, whereas the predictive effect of SES was not significant. Compared to boys, girls were significantly more likely to belong to the Body Other-Appreciation Dominant Group than to the Positive Body Image Group. According to Objectification Theory (Fredrickson and Roberts, 1997), which posits that the sexual objectification of females leads them to internalize an observer's perspective on their own bodies, adolescent girls may be particularly affected. This process can lead them to allocate more cognitive resources to appearance-related societal ideals and to engage in behaviors aimed at approximating those standards. Consequently, although girls may cognitively endorse the notion that “beauty is diverse,” they may struggle to internalize this belief in their self-evaluations, rendering their body image more susceptible to deviation from a healthy state. Regarding the role of BMI, higher BMI was significantly associated with a greater likelihood of belonging to the Body Other-Appreciation Dominant Group relative to the Positive Body Image Group. This may reflect a specific coping mechanism: early adolescents with higher BMI, facing weight-related social pressures, may adopt broader beliefs about beauty as a self-acceptance strategy (Tylka and Iannantuono, 2016), yet struggle to extend this acceptance to their own bodies. Interventions for this subgroup should therefore focus on bridging the gap between other-directed and self-directed body appreciation. In contrast to gender and BMI, SES did not significantly predict membership in the latent body image profiles. This result diverges from some prior research (e.g., van den Berg et al., 2010) but is consistent with other findings (e.g., Wang et al., 2005). Such inconsistency may stem from variations in sociocultural context, the age range of participants, and the measurement tools and assessment methods used for SES. Future research could further explore and refine these aspects.

Furthermore, levels of different maladaptive eating behaviors varied across the identified latent profiles of body image among early adolescents. Regarding restrained eating, both the Body Other-Appreciation Dominant Group and the Moderate Body Image Group exhibited significantly higher levels compared to the Positive Body Image Group. This is consistent with previous research (e.g., Hao et al., 2022; Zarychta et al., 2020) indicating that individuals with greater body dissatisfaction demonstrate a stronger tendency to diet in pursuit of an ideal body shape. Notably, although the Body Other-Appreciation Dominant Group appreciates diverse body types in others, their comparatively lower appreciation and higher dissatisfaction with their own bodies may still drive them to engage in restrained eating as a means to alter their physique. Concerning emotional eating, the Moderate Body Image Group reported significantly higher levels than both the Body Other-Appreciation Dominant Group and the Positive Body Image Group. This finding aligns with previous research (Jackson et al., 2022), which suggests that individuals experiencing a state of co-existing positive and negative body image, specifically a state of “normative ambivalence,” tend to exhibit higher emotional eating than those with a profile characterized by high-positive and low-negative body image. The characteristic “normative ambivalence” of this group may reflect relatively weaker emotion regulation capacities. Consequently, when confronted with stress or negative emotions, these individuals may be more inclined to use eating as an immediate, externalized coping strategy, thereby leading to elevated levels of emotional eating. For external eating, no significant differences were found across the latent body image profiles in this early adolescent sample. This result contradicts some previous studies (e.g., Munkholm et al., 2016; Yang et al., 2022). External eating is primarily driven by environmental food cues (van Strien et al., 1986), and its relationship with body image may be moderated by factors such as cultural context, measurement instruments, and developmental stage. Future research could further examine the roles of cultural background and external environment, or employ longitudinal designs to track how the association between body image and external eating evolves with age.

### 4.1 Implications

These findings have important implications for the prevention and intervention of body image and eating behavior problems among early adolescents. First, given that early adolescence is a critical window for body image development, prevention should be prioritized over treatment-oriented interventions. Even the Positive Body Image Group, which constitutes the largest proportion of the sample, should not be overlooked. Although these adolescents currently exhibit positive body image, early adolescence is a period of rapid change, and without preventive reinforcement, they may be at risk of developing body image concerns during later adolescence. School-based mental health education, group counseling, and campus-wide campaigns that promote positive body image and self-acceptance may help reinforce healthy body image and prevent potential declines during later adolescence. Second, the Body Other-Appreciation Dominant Group may be easily overlooked in practice, as these adolescents appear to hold positive attitudes toward body diversity, yet they struggle to extend the same acceptance to themselves. For this group, interventions fostering body self-compassion and narrowing the gap between other-directed and self-directed body appreciation may be especially beneficial. Third, the Moderate Body Image Group showed elevated emotional eating, suggesting that this subgroup may benefit more from support in emotion regulation than from weight-focused approaches alone. Finally, the gender and BMI differences observed in profile membership highlight the importance of considering individual characteristics when designing prevention programs; interventions tailored to girls and to adolescents with higher BMI may need to pay particular attention to weight stigma and appearance-related societal pressures.

### 4.2 Limitations

Despite the contributions of this study, several limitations should be noted. First, the cross-sectional design precludes strong causal inferences regarding the relationships between different body image profiles and maladaptive eating behaviors. Future research should employ longitudinal or experimental designs to verify these associations. Second, the sample was drawn solely from Heilongjiang Province, China, which may not fully represent the diversity of early adolescents across the nation. Expanding the sample to include regions with varying sociocultural and economic characteristics would enhance the generalizability of the findings. Third, this study focused primarily on demographic factors such as gender, BMI, and SES, without sufficiently examining the potential influences of other critical factors like family interactions, peer influence, and social media use on profile membership. Incorporating these variables in future work would allow for a more nuanced understanding of the influential factors of profile classification. Finally, considering the significant role of family context in shaping body image, future research could also investigate the intergenerational transmission of body image profiles, which would further enrich and refine relevant theoretical frameworks.

## 5 Conclusions

Employing LPA, this study identified three distinct body image profiles among Chinese early adolescents: the Positive Body Image Group, the Body Other-Appreciation Dominant Group, and the Moderate Body Image Group. The analysis confirmed that gender and BMI significantly predicted profile membership, whereas SES did not. In terms of maladaptive eating behaviors, compared with the Positive Body Image Group, the Body Other-Appreciation Dominant Group reported significantly higher levels of restrained eating. Furthermore, the Moderate Body Image Group, which is characterized by the coexistence of both positive and negative body image, exhibited significantly higher levels of both restrained eating and emotional eating relative to the Positive Body Image Group. However, no significant differences were found among the profiles in external eating. These findings indicate that distinct body image profiles are associated with specific patterns of eating-related risks. The results provide an empirical foundation for developing targeted preventive interventions aimed at promoting positive body image and reducing maladaptive eating behaviors among early adolescents.

## Data Availability

The raw data supporting the conclusions of this article will be made available by the authors, without undue reservation.
